# Dental Implants Fatigue as a Possible Failure of Implantologic Treatment: The Importance of Randomness in Fatigue Behaviour

**DOI:** 10.1155/2015/825402

**Published:** 2015-10-25

**Authors:** María Prados-Privado, Juan Carlos Prados-Frutos, Ángel Manchón, Rosa Rojo, Pietro Felice, José Antonio Bea

**Affiliations:** ^1^Department of Stomatology, Rey Juan Carlos University, C/ Tulipán s/n, Móstoles, 28933 Madrid, Spain; ^2^Applied Modelling and Instrumentation Group, Aragón Institute of Engineering Research, University of Zaragoza, C/ Mariano Esquillor s/n, 50018 Zaragoza, Spain; ^3^Department of Biomedical and Neuromotor Sciences, Unit of Periodontology and Implantology, University of Bologna, Via Zamboni 33, 40126 Bologna, Italy; ^4^Aragón Institute of Engineering Research, University of Zaragoza, C/ Mariano Esquillor s/n, 50018 Zaragoza, Spain

## Abstract

*Objective*. To show how random variables concern fatigue behaviour by a probabilistic finite element method. *Methods*. Uncertainties on material properties due to the existence of defects that cause material elastic constant are not the same in the whole dental implant the dimensions of the structural element and load history have a decisive influence on the fatigue process and therefore on the life of a dental implant. In order to measure these uncertainties, we used a method based on Markoff chains, Bogdanoff and Kozin cumulative damage model, and probabilistic finite elements method. *Results*. The results have been obtained by conventional and probabilistic methods. Mathematical models obtained the same result regarding fatigue life; however, the probabilistic model obtained a greater mean life but with more information because of the cumulative probability function. *Conclusions*. The present paper introduces an improved procedure to study fatigue behaviour in order to know statistics of the fatigue life (mean and variance) and its probability of failure (fatigue life *versus* probability of failure).

## 1. Introduction

Fatigue phenomenon is known as the change that appears on materials when cyclic loads are applied. It is possible to find a definition in the report entitled* General Principles for Fatigue Testing of Metals*, which was published in 1964 by The International Organization for Standardization in Switzerland. In this report, fatigue is defined as “a term which applies to changes in properties which can occur in a metallic material due to the repeated application of stresses or strains, although usually this term applies specially to those changes with lead to cracking or failure” [1].

Dental implants have to support many loading cycles during their life; majority of those cycles are produced during physiologic masticatory cycles or parafunctions as bruxism [2]. These additional occlusive forces produce an extra effort in the dental implant and, due to this, fatigue plays a very important role here. Masticatory forces are not constant; they change its value (direction, mean value, etc.), depending on other factors such as the constrains at the joints and bite point, the inclusion, or not, of the periodontal ligament, or the material properties assigned to the cancellous bone tissue; this involves a lot of randomness associated with the fatigue phenomenon. To reduce the unrestrained elements, we have used a method to show how uncertainties concern fatigue life and the probability of failure using a method based on Markoff chains, Bogdanoff and Kozin cumulative damage model, and probabilistic finite elements method [3–5].

Among the factors described as possibly responsible for the failure of implant treatment due to mechanical etiology, it is the clinical phenomenon known as bruxism; the American Academy of Orofacial Pain (AAOP, 2008) defines it such as in [6] “as a movement disorder of the stomatognathic system characterized.” The grinding and clenching of teeth, either day or night, are with a prevalence between 6 and 91% for both sexes in the general population [7], and the age range is between 18 and 49 years [8]. The clinical consequences of bruxism may be different for its different types (awake and sleep), highlighting muscular hypertrophy, tooth wear, tooth fracture, fracture of the restorations or of the implants, sensitivity or pain of the teeth, muscle or joints, and temporomandibular joint disc displacement [7].

Bruxism causes excessive load on dental implants and their superstructures, causing bone loss around implants or even failure of the implant and/or of the implant restoration. Therefore, bruxism is considered a risk factor for implant treatment [9–12].

In order to evaluate the dental implant design propriety, it is necessary to carry out standard fatigue tests with different loads levels, as is explained in ISO 14801. These tests require a lot of time to do all the analysis and high expensive resources because in the practical way to study fatigue it is necessary to analyse a great deal of dental implants until their fracture. The aim of this study was to evaluate the predicted fatigue life of Proclinic dental implant using Markoff chains, Bogdanoff and Kozin cumulative damage model, and the probabilistic finite element method to reduce time and improve dental implants and compare the results with some mathematical models to study fatigue, for example, Goodman or Gerber models [13].

## 2. Materials and Methods

**Figure 1:**
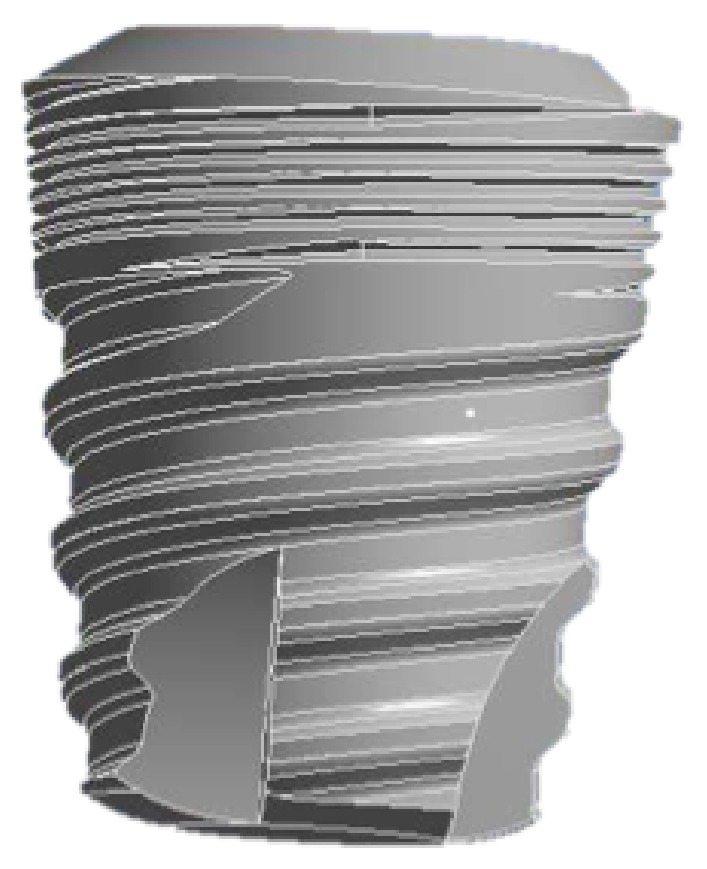
Proclinic dental implant analysed.

In this paper we have analysed a Proclinic dental implant called CON.INT IP887 (Figure 1) which has a hexagonal internal connection, a diameter *D* = 5 mm, and a length *L* = 6 mm. The IGES file used in this study has been provided by Proclinic dental implants, manufactured by Avenir S.L. (Rimini, Italy).

Proclinic dental implant that we have analysed is manufactured of a titanium alloy known as Titanium ELI (Ti6Al4V), with an internal hexagonal connection and with characteristics shown below. This alloy shows excellent performance* in vivo* due to the excellent balance between mechanical, physicochemical, and biofunctional properties [14].

Titanium is a highly biocompatible biomaterial (both* in vitro* and* in vivo*), also bioinert, with a great ability to establish a direct structural and functional connection between ordered and living bone and the surface of the implant [15].

Wrought Ti-6A1-4V is a useful material for surgical implants because of its low modulus, good tensile and fatigue strength, and biological compatibility. It is used for bone screws and for partial and total hip, knee, elbow, jaw, finger, and shoulder replacement joints. Where fatigue properties are not an issue, the cast alloy also has had minor use as an implant product [16].

Geometry in IGES format has been used to generate the finite element mesh employing the software ANSYS CFX (version 14.5, Canonsburg, Pennsylvania, United States). 25427 nodes and 14481 elements compose this mesh. The boundary conditions applied match ISO 14801 test procedure, that is,

- Apical: all DOFs (degrees of freedom, i.e., displacements) restrained,
- Thread: only displacements normal to the surface are allowed,
- Crestal: natural boundary conditions, that is, force, are applied with an orientation of 15 degrees as described.

### 2.1. Conventional Fatigue by Mathematical Models

In this section, we show a quick review about the three more important models in order to remind the reader about those common criteria and, in this way, compare them with the method proposed in this study. Fatigue behaviour is usually studied theoretically by three mathematical models, which were proposed by Goodman, Gerber [13], and Soderberg [17].

**Figure 2:**
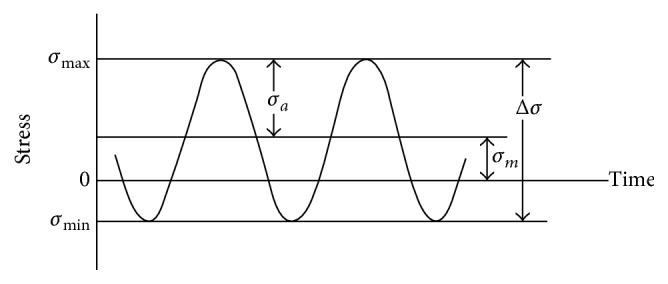
Cyclic loads.

These three relationships, as Meyers and Chawla explain in [18], consider the cyclic load as appears in Figure 2, *σ*_*m*_ being the mean stress and *σ*_*a*_ being the fatigue strength in terms of stress amplitude when *σ*_*m*_ = 0 (stress amplitude).

Mathematically, these three models are expressed as

**Figure 3:**
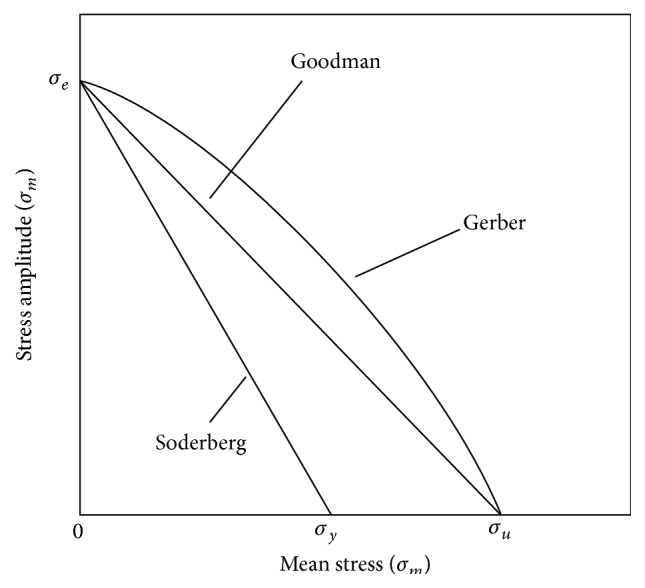
Goodman, Soderberg, and Gerber graphical models.

(1) Goodman:  σa=σe1−σmσu,Gerber:  σa=σe1−σmσu2,Soderberg:  σa=σe1−σmσy,

where *σ*_*u*_ is the ultimate tensile strength and *σ*_*y*_ is the yield strength. Those three expressions can be represented graphically as ASM shows (Figure 3) [14].

The general trend given by the Goodman relation is one of decreasing fatigue life with increasing mean stress for a given level of applied stress. The relation can be plotted to determine the safe cyclic loading of a part; if the coordinate given by the mean stress and the applied stress lies under the curve given by the relation, then the part will survive. If the coordinate is above the curve, then the part will fail for the given stress parameters.

Gerber used a parabolic model and Goodman used a line, which are more conservative [19].

### 2.2. Probabilistic Fatigue Method

Proposal presented here is based on the following questions:

1. Why is it necessary to do a lot of tests with the use, probably excessive, of time and components, uniquely dental implants in this case, and what that implies?
2. What happens to random variables that models discussed above do not consider? Models proposed by Soderberg, Gerber, and Goodman do not take into account that both masticatory forces and material properties do not always have the same value, as can be appreciated in (1).

Most of the fatigue studies are done from the deterministic point of view, while the model used in this study considers the randomness of some variables. The most important difference between the three models explained below and the model used in this work is that in the last one it is possible to take into account defects that can appear in the dental implant and the different mean loads that vary depending on the patient and the masticatory loads.

In order to determine the fatigue life with random variables and to be able to predict the probability of failure for each cycle, authors employed a probabilistic model developed by Bogdanoff and Kozin (B-K), which is based on Markoff chains. To generate the model, we have used the results taken from constant load structural analysis (von Mises stress) employing the commercial software ANSYS CFX.

According to the proposal by Prados-Privado et al. [20] which combines the finite element method and the B-K model it is possible to solve the problem in four steps (one associated with the deterministic problem and three associated with the random variables considered).

**Figure 4:**
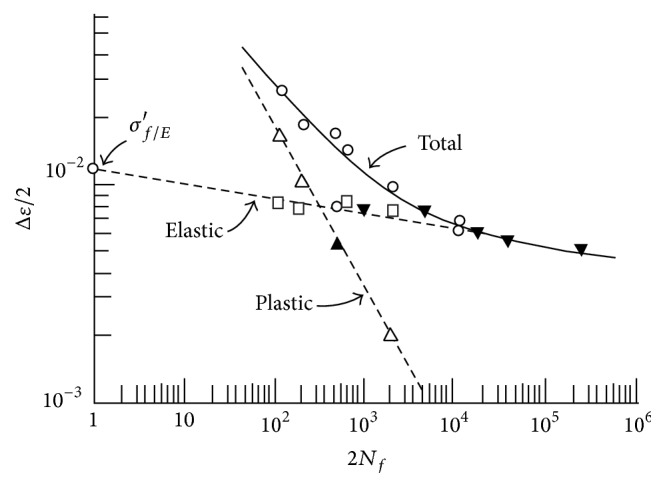
*ε*-*N* curve.

**Figure 5:**
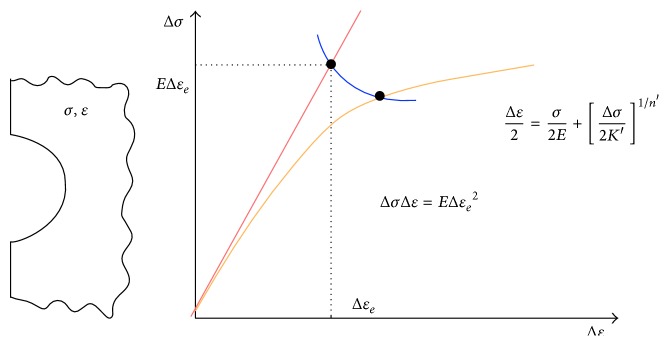
Graphical formulation of Neuber's law.

The main ideas are as follows:

(i) Once the mean and the variance of the fatigue life are known, we are ready to construct the probability transition matrix (PTM). Equations (3) and (4) from the Supplementary Material (available online at http://dx.doi.org/10.1155/2015/825402) allow calculating the parameters needed to construct the PTM.
(ii) To get the mean life, it is necessary to know the *ε*-*N* curve of the material.  Figure 4 shows an example of how *ε*-*N* curve is.
(iii) To get the variance of the fatigue life, it is necessary to know some elastoplastic properties of the material (Neuber's law). In Figure 5 is represented the graphical formulation for Neuber's law, which has been obtain in [21].
(iv) To get the variance of the elastic properties it is necessary to have, first, a stochastic analysis, which is done by ANSYS CFX.

With the idea of a good understanding of the results, here we include a brief description of the probability transition matrix (PTM), as Bogdanoff and Kozin wrote in [3], which has the form shown in the following expression:

(2) $$\begin{array}{c} P=\left(\begin{array}{cccccc} p_{1} & q_{1} & 0 & \cdots & 0 & 0\\ 0 & p_{2} & q_{2} & 0 & \cdots & 0\\ 0 & 0 & p_{3} & q_{3} & \cdots & 0\\ \vdots & \vdots & \vdots & \vdots & \ddots & \vdots \\ 0 & 0 & 0 & \cdots & p_{b-1} & q_{b-1}\\ 0 & 0 & 0 & \cdots & 0 & 1 \end{array}\right), \end{array}$$

where *p*_*j*_ is the probability of remaining in the same stage *j* during damage cycle and *q*_*j*_ is the probability of jumping to the next level that is from the damage state *j* to *j* + 1. Obviously, *p*_*j*_ + *q*_*j*_ = 1 and 0 < *p*_*j*_ < 1. Parameters and expressions required to construct this matrix are included in the Supplementary Material.

This matrix is very important in this study because, without it, it is not possible to calculate our principal aim; in other words, with this matrix we can calculate the dental implant probability of failure.

To obtain the PTM, it is necessary to compute statistics (mean value and variance) of the fatigue life. Bogdanoff and Kozin did it directly from experimental data. The first time that these statistics were numerically evaluated was done by Bea et al. [4]. A choice could be to obtain samples from Monte Carlo Simulation, which is really expensive (it is necessary to compute a hundred or thousand times of fatigue life simulations). We have chosen the PFEM (perturbation method). Instead of generating samples, several Taylor expansions are done around every random variable that affects fatigue life, for all the random fields in continuum mechanics: displacement field, strain field, and stress field. If these fields are known, the fatigue life can be computed [4, 20, 21].

More information about the method used in this paper can be found in the Supplementary Material.

## 3. Results

The main aim of this section is to show the difference between the conventional way and the method we propose here to study fatigue behaviour. In this case, we are applying the method on Proclinic dental implant called CON.INT IP887 shown in Figure 1 using the commercial software Mathematica (version 9, Oxfordshire, United Kingdom).

### 3.1. Material Properties

Table 1 shows the material properties used in this study. These values have been proportioned by Proclinic and obtained from ASM and Kobayashi et al. [22].

### 3.2. Force Bite

In this study, we have used mean and standard deviation on bite force shown in Table 2 and expressed in Newton (SI force units). These values have been obtained as a mean on values that Clark and Carter used in their study [23]. Values in Table 2 are a statistical analysis about the bite force in both genders without any health and dentist problems done with their molars.

### 3.3. Analysis

The most important results gained by employing the probabilistic model are shown in Table 3. From a conventional way, the same result is obtained. This means that, in this case, this implant should have the same life independently of the method used. The probabilistic model proposed here gets a bigger life but, however, it is more precise because this model provides the mean fatigue life, the variance for this life, and a cumulative probability function as Figure 7 shows.

It should be pointed out that there is no variance on the three first models because they are deterministic; because of that variance is only applicable in the probabilistic model.

**Figure 6:**
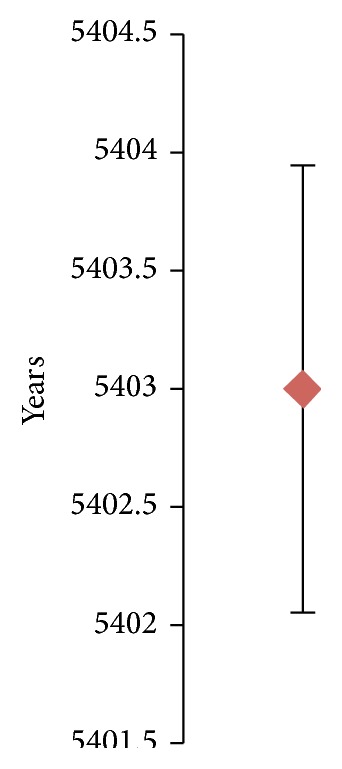
Minimum and maximum life.

Figure 6 shows the life range in which the Proclinic dental implant analysed is going to be.

Here we find the first main difference between the results obtained by conventional fatigue and the probabilistic model proposed in this paper. The results show a big difference, but it is necessary to understand that the result gained by conventional fatigue means that the fatigue life is going to be bigger than 48 years but it does not say anything about the probability of failure.

**Figure 7:**
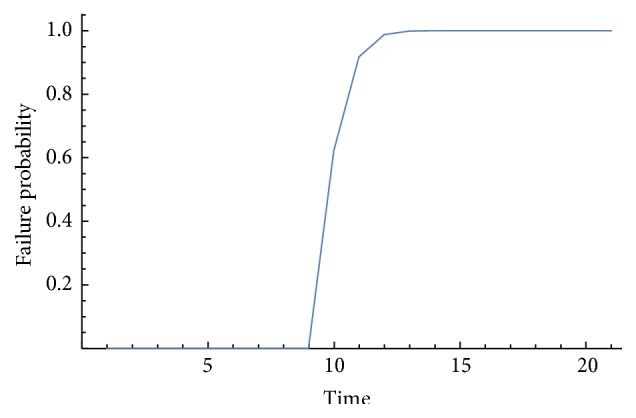
Cumulative probability function.

Values obtained to get Figure 7 are those shown in Table 4. In order to get a good graph to show with detail what happens on implants, it was necessary to change the temporal axes, so, column “time” is the value which has to be multiplied by the number of cycles to know the probability of failure associated with each cycle.

Figure 7 has the following meaning:

1. Cumulative probability function relates probability of failure with number of cycles.
2. Until time 9, the probability of failure is zero, so, that means that nothing is waiting to occur until this time.
3. If we get the probability of failure associated with the mean life obtained and shown in Table 3, we discover that it is very close to 50%.
4. Minimum life obtained with the method proposed here has a zero probability of failure.
5. Maximum life obtained has a 60% of probability of failure associated, approximately.

## 4. Discussion and Conclusion

In this paper we present the application of a probabilistic methodology for dental implants, taking into account the variability in loads, fatigue behaviour, and design aspects and comparing results with deterministic fatigue. This probabilistic methodology, based on Markoff chains, was first applied by Bea et al. [4, 21], to metal fatigue including separately crack nucleation and crack growth stage.

Uncertainties between mastication habits among different patients imply that random loads are applied to the implants and since this must be taken into account in the model from the very beginning it can be done using Markoff chains. Most FE studies on dental implants are static analyses [24–29].

This methodology allows us to keep in mind different load blocks with different amplitudes, considering their sequential effects. These sequential effects achieve more realistic and confident results and they are not used in this study because the aim is to present the main difference between the conventional way of studying fatigue behaviour and a probabilistic method.

Main differences between the conventional way and the method we have proposed here are as follows:

- First of all, conventional fatigue studies do not take into account uncertainness that material or loads can have.
- With the probabilistic model, it is possible to know the mean fatigue life, the variance, and, what is more important, the probability of failure for each cycle.
- In this case, results are the same for the three models used to study conventional fatigue life.
- As Figure 7 shows, it is possible to ensure that the minimum fatigue life for this dental implant is 5388 years, because, below this time, the probability of failure is zero.
- There is a big difference in fatigue life obtained by deterministic or probabilistic models. This means that with the probabilistic method we are able to know with more detail the behaviour that Proclinic dental implant analysed here is going to have, keeping in mind always that this case is also including random variables, which introduced more confidence in the model and results obtained.

## Supplementary Material

In where it is explained the mathematical questions about the model we propose here. With this Supplementary Material, it is possible to construct the model used in this study.

## Figures and Tables

**Table 1:** Titanium alloy properties employed.

Emblem	*E* [GPa]	*b* [—]	*c* [—]	*σ*_*f*′_ [Pa]	*ε*_*f*′_ [—]	*σ*_*y*_ [MPa]	*σ*_*u*_ [MPa]
Value	114	−0,018	−0,026	1,4 · 10^9^	0,0186	828	895

*E*: modulus of elasticity (whose units are Pascal, SI pressure units).

*b*: fatigue resistance exponent (nondimensional).

*c*: fatigue ductility exponent (nondimensional).

*σ*_*f*′_: fatigue resistance coefficient (Pa).

*ε*_*f*′_: fatigue ductility coefficient (nondimensional).

*σ*_*y*_: tensile yield strength (Pa).

*σ*_*u*_: tensile ultimate strength (Pa).

**Table 2:** Bite force: mean and standard deviation.

Mean [*N*]	Standard deviation [*N*]
583,49	72,6

**Table 3:** Fatigue results for Proclinic dental implant analysed.

	Goodman	Soderberg	Gerber	Probabilistic model
Life	>1,7 · 10^9^ cycles	>1,7 · 10^9^ cycles	>1,7 · 10^9^ cycles	1,18 · 10^11^ cycles
	(>48,7 years)	(>48,7 years)	(>48,7 years)	(5403 years)

Variance		Not applicable		4,28 · 10^14^
				(0.89 years^2^)

**Table 4:** PTM parameters.

Matrix dimension	Time	*p*	*q*
3500	1.54	0.000106675	0.999893

## Abbreviations

ASM: American Society of Metals
B-K: Bogdanoff and Kozin
PTM: Probability transition matrix
FE: Finite element.
