# Supplementary material for: Dental Implants Fatigue as a Possible Failure of Implantologic Treatment: The Importance of Randomness in Fatigue Behaviour
Source: Biomed Res Int. 2015 Oct 25;2015:825402. doi: 10.1155/2015/825402 (PMC4637060; doi:10.1155/2015/825402)
Supplement: Supplementary file 1 — In where it is explained the mathematical questions about the model we propose here. With this Supplementary Material, it is possible to construct the model used in this study. [file 825402.f1.pdf]

APPENDIX

Mathematical questions about the probabilistic fatigue method proposed in this work are explained in this Appendix.

Construction of the cumulative damage B-K model

The B-K model is based on some assumptions. The most important are the following:

1. There exist repetitive **"damage cycles"** of constant severity, DC from now on. There may be several blocks of constant load, each one will have associated a probability transition matrix.
2. The damage level are discrete (1, 2, …, j, …, b). The last level, b, is considered as a **"failure stage"**.
3. The cumulative damage in a DC depends only on that specific DC and the damage level at the beginning of that DC.
4. The damage level in one DC increases from DC_i to the DC_{i+1} or remains in the initial DC_i, being impossible to skip one or more damage states.

The damage process defined by the above assumption can be considered as a Markoff process because they are defined as discrete (discrete damage cycles in the first two hypothesis) and conform to the main property of a Markoff process (third hypothesis). Also, if you define that the probability of jumping from one level to the next level of damage remains constant throughout the degradation process, we will face a stationary Markoff process. The second hypothesis sets that the failure appears when we get the last damage level, b, so this stage will be the absorption stage. The others levels are stationary. Also, the damage is considered irreversible: when a certain damage level is reached, it is not possible to come back to lower damage levels [3].

We can write the above assumptions immediately

$$\boldsymbol{p}_t = \boldsymbol{p}_0 \boldsymbol{P}^t \quad t = 0,1,2 \ldots \tag{1}$$

where p_t is the probability of reach each stage 1,2,…,b in the time t, p_0 the initial distribution of the different damage level at t=0 and **P** is the probability transition matrix. By the first assumption, all damage cycles have the same severity and, as we have considered a one-step B-K model, the probability matrix transition has the form shown in (2).

$$
\boldsymbol{P} = \begin{pmatrix} p_1 & q_1 & 0 & \cdots & 0 & 0 \\ 0 & p_2 & q_2 & 0 & \cdots & 0 \\ 0 & 0 & p_3 & q_3 & \cdots & 0 \\ \vdots & \vdots & \vdots & \vdots & \ddots & \vdots \\ 0 & 0 & 0 & \cdots & p_{b-1} & q_{b-1} \\ 0 & 0 & 0 & \cdots & 0 & 1 \end{pmatrix} \quad (2)
$$

As Bea explains in [4], the mean and the variance of the fatigue life can be obtained in function of the parameters p, q and r, as shown in the expressions (3) and (4).

$$
E[N_f] = \sum_{j=1}^{b-1} (1 + r_j) \quad (3)
$$

$$
var[N_f] = \sum_{j=1}^{b-1} r_j(1 + r_j) \quad (4)
$$

With:

$$
r_j = \frac{p_j}{q_j} = \frac{1}{q_j} - 1 \text{ and } p_j = \frac{r_j}{1+r_j} \quad (5)
$$

If the mean and the variance of the fatigue life are known, it is possible to built the B-K model from expressions (3) and (4).

Random formulation of Coffin and Basquin-Manson expressions

Equation (6) summarizes the deterministic formulation for the fatigue life in the nucleation stage.

$$
\frac{\Delta\varepsilon_{ep}}{2} = \frac{\sigma_f'}{E}(2N_f)^b + \varepsilon_f'(2N_f)^c \quad (6)
$$

where:

$\Delta\varepsilon_{ep}$: elastic-plastic strain amplitude

σ_f': fatigue strength coefficient

b: fatigue strength exponent

ε_f': fatigue ductility coefficient

c: fatigue ductility exponent

E: Young modulus

N_f: fatigue life

The probabilistic finite element method

The perturbation method was proposed by Hisada and Nakagiri in 1981, [5]. The aim is to obtain the response random fields with the first order Taylor expansions, thus it will be necessary to obtain, firstly, the sensitivities. To do this, it is require taking derivative of the expression (7) respect to random variables, expression (8).

$$\boldsymbol{f} = K\boldsymbol{u} \tag{7}$$

$$\frac{\partial \boldsymbol{f}}{\partial \alpha_i} = \boldsymbol{u}\frac{\partial K}{\partial \alpha_i} + K\frac{\partial \boldsymbol{u}}{\partial \alpha_i} \tag{8}$$

If we solve expression (8), for each random variable, it is possible to obtain the sensitivities for the vector $\boldsymbol{u}$, $\frac{\partial \boldsymbol{u}}{\partial \alpha_i}$. Thus, it will be inevitable to calculate the sensitivities for each random variable.
